# Distinguishing Acute Rheumatic Fever From Post-streptococcal Reactive Arthritis

**DOI:** 10.7759/cureus.55739

**Published:** 2024-03-07

**Authors:** Su Hyun Jeong, Nishitha Shekhar, Neelesh Mutyala, Omar Canaday

**Affiliations:** 1 Internal Medicine, University of Nevada Reno School of Medicine, Reno, USA; 2 Internal Medicine, McGovern Medical School, Houston, USA; 3 Medicine, Veterans Affairs (VA) Sierra Nevada Health Care System, Reno, USA

**Keywords:** group a streptococcal bacteremia, jones criteria, rheumatology, poststreptococcal reactive arthritis, acute rheumatic fever

## Abstract

We report an initial episode of post-streptococcal reactive arthritis (PRSA) in a 61-year-old male with group A streptococcal (GAS) bacteremia. The disease is commonly reported in young children and young adults. Additionally, this patient exemplifies the nonlinear boundaries of acute rheumatic fever (ARF) and PRSA, bringing into question whether they are truly distinct disease entities. These two conditions oftentimes present in similar fashions, making it difficult for clinicians to determine a specific diagnosis. We highlight the importance of recognizing ARF versus PRSA as an incorrect diagnosis can lead to the development of harmful complications including rheumatic heart disease (RHD).

## Introduction

Acute rheumatic fever (ARF) is an inflammatory disease that can follow an inadequately treated group A streptococcal (GAS) infection after two to four weeks. The disease is predominantly seen in children and is uncommon in the United States, with less than two cases per 100,000 school-aged children each year [1]. The pathogenesis is incompletely understood but believed to involve an autoimmune response to a cross-reactive GAS antigen [2]. Diagnosis is determined clinically based on the revised 2015 Jones criteria but can still be easily missed, resulting in Jaccoud arthropathy or rheumatic heart disease (RHD) [3].

Meanwhile, post-streptococcal reactive arthritis (PSRA) is an inflammatory arthritis of one or more joints associated with a recent GAS infection in someone who does not satisfy the Jones criteria. It is generally considered a separate condition from ARF with a different approach to management. For example, only ARF requires antibiotic prophylaxis to prevent recurrence. Despite these differences, there is still debate regarding whether these two diseases are fundamentally different or a continuum of the same condition. The distinction in adults is less clear as most studies focused on children.

This clinical vignette demonstrates the overlapping features between ARF and PSRA and the challenges involved in determining a diagnosis.

## Case presentation

A 61-year-old male with a past medical history of gastroesophageal reflux disease (GERD), allergic rhinitis, and erectile dysfunction presented with a weeklong history of diffuse body aches and joint pain. Fifteen days ago, he reported a sore throat and fevers for which he had presented to an outside facility. There, he was found to be GAS-positive with a temperature of 103.1 degrees Fahrenheit. He was diagnosed with GAS pharyngitis and discharged with amoxicillin. Reportedly, the patient was unable to pick up his antibiotics and rapidly deteriorated so that he was unable to leave his home.

During this admission, a physical exam demonstrated polyarthritis of all his left fingers, with swelling and erythema of the metacarpal phalanges. There was also erythema and swelling of the proximal and distal interphalangeal joints of his left index finger. He had concomitant polyarthralgia without erythema or swelling of the right hand, right knee, right wrist, and right ankle. The patient continued to endorse pharyngitis. Vitals include a temperature of 98.5 degrees Fahrenheit, heart rate of 78 beats per minute, respiratory rate of 18 breaths per minute, blood pressure of 131/80 mmHg, and oxygen saturation of 98% on room air. Pertinent labs include an erythrocyte sedimentation rate (ESR) of 95 mm/hr, c-reactive protein (CRP) of 30.2 mg/dL, white blood cell count (WBC) of 21.21 K/uL, and blood cultures positive for GAS. Antinuclear antibody (ANA), rheumatoid factor (RF), and complement levels were within normal limits. The complete lab work is listed in Table 1.

**Table 1:** Lab work during hospitalization CO2: Carbon dioxide; AST: Aspartate transaminase; ALT: Alanine transaminase; HLA: Human leukocyte antigen

	Day of admission	Day of discharge	Reference range
C-reactive protein	30.20	3.20	0.00-5.00 mg/dL
Erythrocyte sedimentation rate	95.00		0.00-20.00 mm/hr
Sodium	127.00	136.00	136.00-146.00 mmol/L
Potassium	3.00	4.50	3.60-5.30 mmol/L
Chloride	88.00	102.00	102.00-114.00 mmol/L
CO2	25.00	28.00	24.00-32.00 mmol/L
Glucose	163.00	97.00	70.00-109.00 mg/dL
Urea nitrogen	19.00	20.00	8.00-25.00 mg/dL
Creatinine, serum	0.90	0.80	0.70-1.30 mg/dL
Uric acid	4.10		3.70-8.20 mg/dL
AST	65.00	14.00	13.00-33.00 U/L
ALT	48.00	22.00	7.00-31.00 U/L
Total bilirubin	1.20	0.30	0.40-1.30 mg/dL
White blood cell count	21.21	7.41	4.00-11.00 K/uL
Red blood cell count	4.13	4.25	4.70-6.10 M/uL
Hemoglobin	11.90	12.40	14.00-18.00 g/dL
Hematocrit	35.30	37.60	39.00-52.50%
Platelet	339.00	577.00	128.00-412.00 K/uL
Creatine phosphokinase	273.00		0.00-243.00 U/L
Lactic acid	2.40		0.60-2.70 mmol/L
Antinuclear antibody	NEG		NEG
Rheumatoid factor	12.00		0.00-14.00 lU/mL
Thyroid-stimulating hormone	0.83		0.35-5.00 ulU/mL
Double-strand DNA antibody	<1:10		NEG <1:10
Complement C3	185.30		82.00-193.00 mg/dL
Complement C4	58.80		10.00-40.00 mg/dL
HLA B-27	NEG		NEG

A transthoracic echocardiogram (TTE) revealed a small, mobile mass on the ventricular surface of the anterior mitral valve leaflet. This was initially concerning for endocarditis versus rheumatic heart disease, but a transesophageal echocardiogram (TEE) two days later showed no evidence of vegetation on the heart valves. X-rays of the left hand revealed mild osteoarthritic changes in the distal interphalangeal joints while those of the right hand were unremarkable. X-rays of the right ankle and knee were negative for signs of inflammation or fractures. He also had a lumbar X-ray which ruled out spondyloarthropathy.

Treatment started with empiric ampicillin-sulbactam and naproxen, which improved the patient’s sore throat. Antibiotics were changed to penicillin and later changed to ceftriaxone after the TEE was performed, with a total duration of 14 days. A decision to complete an antibiotic course inpatient was made due to the patient’s socioeconomic barriers. Management of his musculoskeletal pain was more modest. Symptom relief with naproxen was minimal, so a five-day prednisone course was added on day three of the admission. He experienced re-exacerbation of joint pain on his fingers prompting a change of his naproxen to celecoxib. Eventually, the patient had two negative blood cultures and near complete resolution of his musculoskeletal symptoms. He was discharged after 14 days with celecoxib and outpatient follow-up to receive prophylactic penicillin G benzathine. Notably, he re-presented to the emergency department with a relapse in joint pains after cessation of celecoxib and was prescribed a longer course.

## Discussion

Most cases of PSRA are reported bimodally in children between eight and 14 years old and adults between 21 and 37 years old [4]. The typical age for incidences of ARF is children between five and 14 years old [5]. For ARF, this may be due to age-related risk factors such as an underdeveloped immune response [6]. We assume this is also the case when children develop PSRA, but it is unclear why we see an increased incidence in young adults. Regardless, our patient was 61 years old when he experienced his first episode. Both illnesses oftentimes present in resource-poor areas with inadequate treatment of infections and this patient was no exception. He was unemployed and living in a shelter, which likely contributed to his medical nonadherence.

The diagnosis of ARF is based on the Jones criteria, which were revised in 2015 [7]. Two classification systems exist based on whether someone is low risk or medium to high risk. An initial episode of ARF requires two major criteria or one major plus two minor criteria. A recurrent episode of ARF requires two major criteria, one major and two minor criteria, or three minor criteria to be met. Our patient is in the low-risk population and did not satisfy the Jones criteria by meeting the major criterion of polyarthritis and one minor criterion of elevated inflammatory markers. Although he had concomitant polyarthralgia, joint manifestations can only be counted once as either a major or minor criterion. Major and minor criteria for both low and medium to high-risk populations are listed in Tables 2, 3 below.

**Table 2:** Major criteria for the diagnosis of acute rheumatic fever

Low-risk populations	Moderate and high-risk populations
Carditis: clinical and/or subclinical	Carditis: clinical and/or subclinical
Polyarthritis	Monoarthritis or polyarthritis, polyarthralgia
Chorea	Chorea
Erythema marginatum	Erythema marginatum
Subcutaneous nodules	Subcutaneous nodules

**Table 3:** Minor criteria for the diagnosis of acute rheumatic fever ESR: Erythrocyte sedimentation rate; CRP: C-reactive protein

Low-risk populations	Moderate and high-risk populations
Polyarthralgia	Monoarthralgia
Fever	Fever
ESR >= 60 mm in the first hour and/or CRP >= 3.0 mg/dL	ESR >= 30 mm in the first hour and/or CRP >= 3.0 mg/dL
Prolonged PR interval, after accounting for age variability (unless carditis is a major criterion)	Prolonged PR interval, after accounting for age variability (unless carditis is a major criterion)

Of note, we did not include fever as the patient was afebrile on admission. We also did not count the fevers he reported before arrival. Had fever been included, he would have satisfied the Jones Criteria. The patient was afebrile despite cultures growing GAS, which is unusual but observed in the literature. One report found that 12% of its nonfebrile participants had positive blood cultures [8]. This same study attributes the presence of bacteremia to disease acuity and lack of treatment. Our patient did not complete his antibiotic course, but receiving initial treatment may have curbed the development of symptoms commonly seen in bacteremia.

The Jones criteria is limited with a modest specificity of 62% but has a high sensitivity of 93% [9]. Given this high sensitivity, we presume he did not have ARF. However, the same source further concludes that in endemic populations strict application of Jones criteria can lead to an underdiagnosis of ARF. Beyond the Jones criteria, reports of immunologic differences can aid in the diagnosis of ARF versus PSRA. One study found patients with ARF had significantly higher ESR and CRP levels [10]. Our patient on admission had an elevated ESR of 95 mm/hr and CRP of 30.2 mg/dL. Another paper discovered increased expression of the human leukocyte antigen (HLA)-B27 allele in PSRA but not in ARF, hinting at a different pathogenesis [11-12]. This patient tested negative for HLA-B27, which again contradicts his diagnosis of PSRA. Although this was not confirmed with our patient, a third study reported a larger significant difference in B cells expressing the D8/17 antigen versus control subjects when comparing ARF and PSRA [13].

The clinical course was more akin to that of PSRA. ARF typically has an onset of 10-28 days from initial GAS infection compared to an average disease onset of 7-10 days for PSRA [14]. Disease onset for our patient occurred eight days after the development of GAS pharyngitis. Cardiac manifestations are also unlikely in PSRA, while carditis is found to occur in 50-70% of initial episodes of ARF [15]. Our patient also lacked cardiac symptoms and his TEE was unremarkable for inflammatory changes. Furthermore, people with PSRA have longer durations of arthritic symptoms and a poorer response to anti-inflammatory medications than those with ARF [10]. This was seen in our case as there was an extended course of joint pains that re-occurred after ceasing anti-inflammatory medications. The patient experienced an incremental improvement in his arthritis and arthralgia using non-steroidal anti-inflammatory medications, which additionally prompted a concurrent short course of steroids for further symptom relief.

## Conclusions

Our 61-year-old patient with GAS bacteremia was diagnosed with an initial bout of PSRA. He failed to meet diagnostic criteria with one major criterion of polyarthritis and only one minor criterion of elevated inflammatory markers. The diagnosis was not delayed, and the patient was without any serious complications. The case highlights the challenges of differentiating ARF from PSRA. Even with the Jones criteria, it can be hard to definitively discern one disease from the other. Additionally, the patient did not have a fever on presentation but was febrile days prior. Had this patient not taken any antibiotics, he may have presented with a fever and been diagnosed with ARF. These minutiae can result in major changes in diagnosis and disease management. In conclusion, there is still much to elucidate regarding the distinction between ARF and PSRA. The Jones criteria is the gold standard but is not without its limitations. A failure to distinguish ARF from PSRA can lead to adverse consequences, which include a higher rate of recurrence and the development of RHD.
